# Assembly and comparative analysis of the complete mitochondrial genome of *Viburnum chinshanense*

**DOI:** 10.1186/s12870-023-04493-4

**Published:** 2023-10-11

**Authors:** Haoxiang Zhu, Yuanyu Shan, Jingling Li, Xue Zhang, Jie Yu, Haiyang Wang

**Affiliations:** 1 College of Horticulture and Landscape Architecture, Southwest University, Chongqing, 400716 China; 2 Key Laboratory of Agricultural Biosafety and Green Production of Upper Yangtze River (Ministry of Education), Southwest University, Chongqing, 400715 China

**Keywords:** *Viburnum chinshanense*, Mitochondrial genome, RNA editing; MTPT, Dipsacales

## Abstract

**Background:**

*Viburnum chinshanense* is an endemic species found exclusively in the North-Central and South-Central regions of China. This species is a lush garden ornamental tree and is extensively utilized for vegetation restoration in rocky desertification areas.

**Results:**

In this study, we obtained 13.96 Gb of Oxford Nanopore data for the whole genome, and subsequently, by combining Illumina short-reads, we successfully assembled the complete mitochondrial genome (mitogenome) of the *V. chinshanense* using a hybrid assembly strategy. The assembled genome can be described as a circular genome. The total length of the *V. chinshanense* mitogenome measures 643,971 bp, with a GC content of 46.18%. Our annotation efforts have revealed a total of 39 protein-coding genes (PCGs), 28 tRNA genes, and 3 rRNA genes within the *V. chinshanense* mitogenome. The analysis of repeated elements has identified 212 SSRs, 19 long tandem repeat elements, and 325 pairs of dispersed repeats in the *V. chinshanense* mitogenome. Additionally, we have investigated mitochondrial plastid DNAs (MTPTs) and identified 21 MTPTs within the mitogenome and plastidial genome. These MTPTs collectively span a length of 9,902 bp, accounting for 1.54% of the mitogenome. Moreover, employing Deepred-mt, we have confidently predicted 623 C to U RNA editing sites across the 39 protein-coding genes. Furthermore, extensive genomic rearrangements have been observed between *V. chinshanense* and the mitogenomes of related species. Interestingly, we have also identified a bacterial-derived tRNA gene (*trnC-GCA*) in the *V. chinshanense* mitogenome. Lastly, we have inferred the phylogenetic relationships of *V. chinshanense* with other angiosperms based on mitochondrial PCGs.

**Conclusions:**

This study marks the first report of a mitogenome from the *Viburnum* genus, offering a valuable genomic resource for exploring the evolution of mitogenomes within the Dipsacales order.

**Supplementary Information:**

The online version contains supplementary material available at 10.1186/s12870-023-04493-4.

## Introduction

Viburnum is a large and diverse genus of flowering shrubs and trees, comprising nearly 200 species worldwide [1]. This genus is renowned for its ornamental value, as many of its species are prized for their attractive flowers, foliage, and berries. Moreover, certain *Viburnum* species possess medicinal properties and are utilized in traditional medicine. For instance, *Viburnum taitoense* Hayata has a long history of folk medicine usage among minority communities in Southwestern China [2]. Similarly, *Viburnum opulus* L. is highly valued as a decorative, medicinal, and food plant, traditionally employed to alleviate various ailments such as cough, colds, tuberculosis, rheumatic aches, ulcers, stomach and kidney problems, among others [3]. Among the *Viburnum* species, *Viburnum chinshanense* Graebn is native to the North-Central and South-Central regions of China. Like most *Viburnum* species, *V. chinshanense* features lush white flowers, vibrant red fruits, and serves as an excellent choice for garden ornamentation. Additionally, its fruits are edible and can be used as fodder. Phytochemical analysis of *V. chinshanense* has revealed that the above-ground plant parts primarily contain six iridoid glucosides [4].

As a genus with significant species diversity and economic importance, there is limited information available on the genomes of *Viburnum*. Previous research has predominantly focused on the study of plastidial genomes in *Viburnum*. The plastidial genomes is an organelle found in plants and some protists, which has its own genome. Its genetic information primarily encodes genes involved in photosynthesis and other chloroplast-specific functions. The plastidial genomes is relatively small and highly conserved, making it commonly used for phylogenetic studies to understand the origin, evolutionary relationships, and taxonomic classification of species [5, 6]. Ran [7] reported the complete plastidial genomes of 21 Chinese *Viburnum* plants and conducted phylogenetic analyses at the whole-plastome level. Subsequently, the plastidial genomes of several other *Viburnum* plants, such as *V. burejaeticum* [8], *V. sargentii* [9], and *V. odoratissimum* [10] have been published. However, to date, there have been no reports on the nuclear or mitogenome of this genus.

Mitochondria play a vital role in energy synthesis and conversion for various cellular physiological activities [11, 12], making them crucial for plant growth and development. They convert biomass energy into chemical energy through phosphorylation and are involved in cell division, differentiation, and apoptosis [13–15]. According to the endosymbiotic theory, mitochondria originated from the endosymbiosis of alpha-bacteria within archaea-derived host cells, eventually evolving into organelles of eukaryotic cells [16]. Mitochondria are unique organelles independent of the nucleus and possess their own genome, which is inherited in a haploid, asexual, and maternal manner [17]. Although mitogenomes, like plastidial genomes, are maternally inherited and contain fewer genes, there are notable evolutionary differences between the two. Plant mitogenomes exhibit substantial variation in size, ranging from 60 kb to over 11 Mb across different species, a much wider range than observed in plastid genomes. [18, 19]. Additionally, while most plant plastidial genomes have a circular structure, higher plant mitogenomes exhibit circular, linear, and even complex branching and reticular structures [20–22]. Furthermore, plant mitogenomes have a higher mutation rate compared to nuclear genomes due to the absence of efficient DNA repair systems [23, 24]. This higher mutation rate contributes to rearrangements, duplications, and the generation of subgenomic configurations within the mitogenome. In contrast, plastidial genomes are relatively stable, except for *Selaginella* species, which also lack certain key DNA-repair enzyme systems [25]. Moreover, some plant mitogenomes have acquired genes through horizontal gene transfer from other organisms. This phenomenon is particularly common in higher plants, where they acquire several plastid sequences from their chloroplast neighbors. This process has occurred over an extended period and is likely ongoing [26–28].

Currently, the availability of mitogenome resources for the Dipsacales order is extremely limited. Only four species can be found in the NCBI nucleotide database (https://www.ncbi.nlm.nih.gov/nuccore/), namely *Sambucus nigra* (OX422225.1), *Triosteum pinnatifidum* (NC_064333.1), *Lonicera japonica* (MZ504724.1), and *Valeriana dageletiana* (MW560898.1). In comparison to the abundance of plastidial genome resources, the scarcity of mitogenome resources significantly hampers our research on the evolution of mitogenomes in this group.

In this study, we performed the following tasks: (1) assembled and annotated the mitogenome and plastidial genome of *V. chinshanense*, (2) using PCR experiments to verify the correctness of the mitogenome assembly, (3) analyzed the repeated elements and mitochondrial plastid sequences (MTPTs), (4) predicted the RNA editing sites in mitochondrial PCGs and analyzed the gain and loss of genes. Lastly, we (5) inferred the phylogenetic relationships of *V. chinshanense* and other angiosperms based on mitochondrial PCGs. Our experiment reported the mitogenome of *V. chinshanense* for the first time, provided a theoretical basis for the organelles study of *V. chinshanense*, and enriched the mitogenome resource of the Dipsacales order.

## Results

### Genomic structure of the *V. chinshanense* mitogenome

**Fig. 1:**
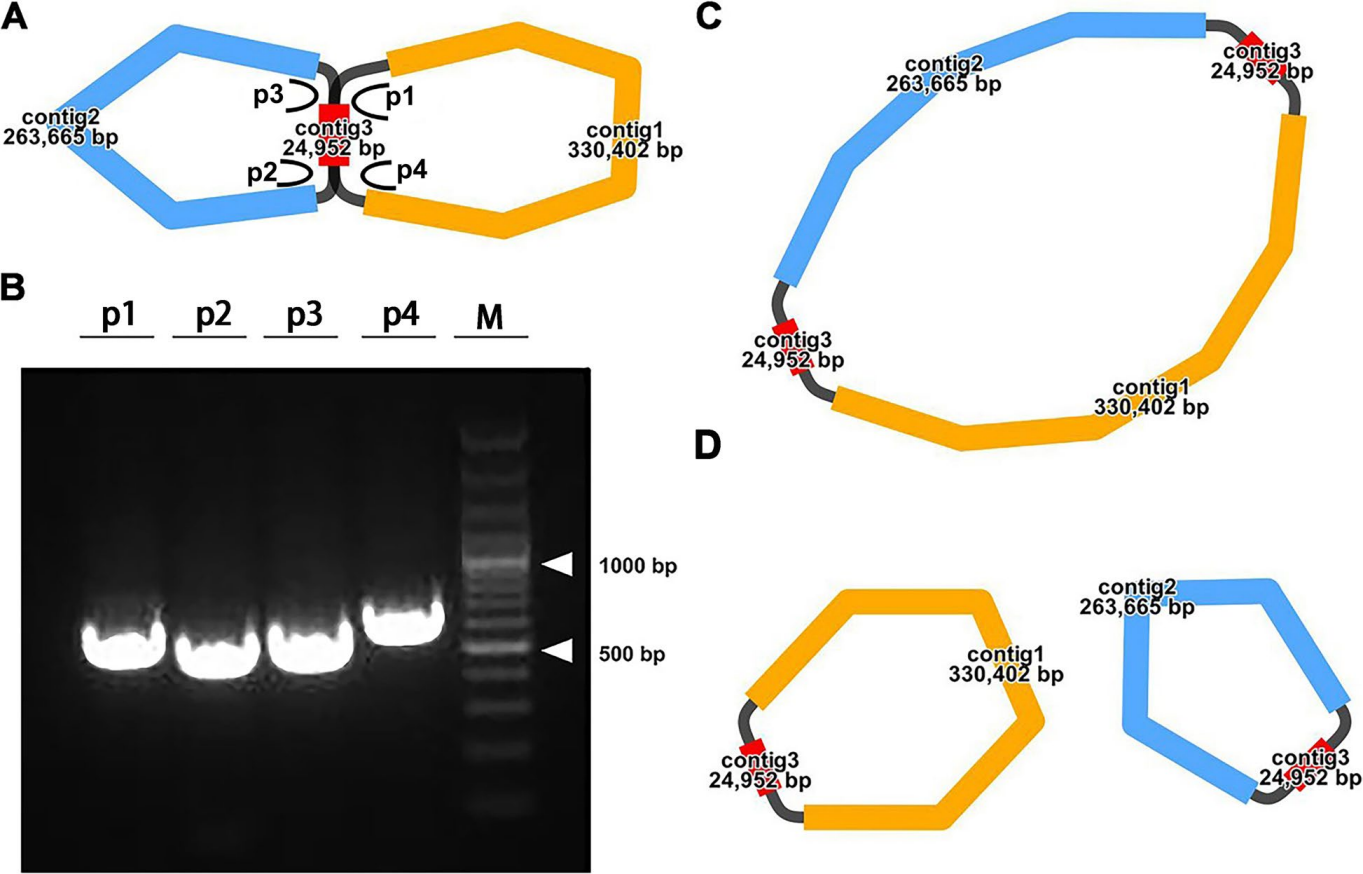
The graphic assembly of *V. chinshanense* mitogenome. **A.** The graphic mitogenome consists of three contigs with different lengths, and they connected to each other. The length of the three contigs are 330,402 bp, 263,665 bp, and 24,952 bp, respectively. Contig 3 is the potential repetitive sequence. Panel **B** and **C** represent the potential master circle structure and the potential alternative structure mediated by contig 3. Panel **D** represents the electropherogram of four paths

**Fig. 2:**
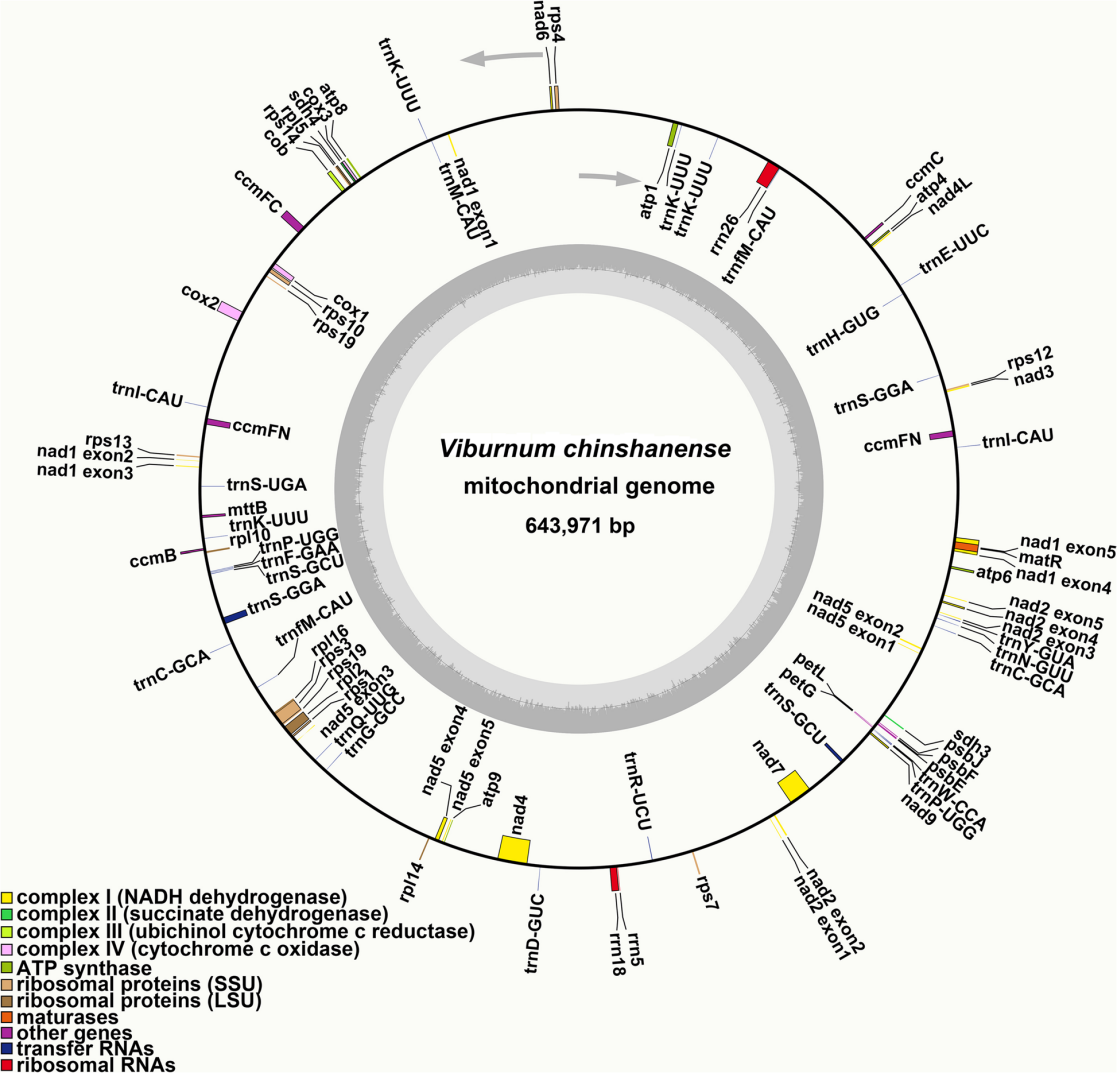
The mitogenome map of *V. chinshanense*. The figure shows the master circle of *V. chinshanense* mitogenome. Genes transcript clockwise or counter-clockwise strands are drawn on the upper or lower of the circles, respectively. Genes belonging to different functional groups are color-coded

We utilized the Flye assembler (v.2.9.1-b1780) to assemble the mitogenome of *V. chinshanense*. Subsequently, the Bandage software was employed to visualize the assembled mitogenome graphically. The assembly graph consisted of three nodes, as depicted in Fig. 1A. Each node represented an assembled contig, and they exhibited an overlapping region along the connecting lines. Notably, contig3 displayed characteristics indicative of a potential repeating sequence. Based on this observation, we proposed two possible genomic configurations: (1) a master circular structure encompassing all three contigs (Fig. 1C), and (2) an alternative configuration wherein contig1 and contig2 form two independent circles with contig3, respectively (Fig. 1D). However, as the length of contig3 exceeded the reach of our sequencing long reads, there were no reads available to directly span this repeating region. Consequently, determining the true structure proved challenging. To confirm the accuracy of the two potential genomic configurations, we conducted PCR experiments followed by Sanger sequencing. Four pairs of primers were utilized to validate the correctness of path p1, p2, p3, and p4, respectively (Fig. 1A). The PCR amplification results displayed bands of the expected sizes (Fig. 1B), and the Sanger sequencing results (Figure S1) confirmed the correctness of the two potential genomic configurations. The untreated electropherogram is presented in Figure S2.

For the master circle of *V. chinshanense* mitogenome, the total length is 643,971 bp, with a GC content of 46.18%. For the alternative conformation, the length of two small circular genome is 355,354 bp and 288,617 bp, respectively. For the purpose of characterization, we have adopted the structure of the master circle in our analysis.

### Gene content of the *V. chinshanense* mitogenome

The *V. chinshanense* mitogenome comprises a total of 39 unique protein-coding genes (PCGs), which can be categorized into 24 core genes and 15 variable genes (Table 1). The 24 core genes consist of five ATP synthase genes (*atp1*, *atp4*, *atp6*, *atp8*, and *atp9*), four cytochrome *c* biogenesis genes (*ccmB*, *ccmC*, *ccmFC*, and *ccmFN*), nine NADH dehydrogenase genes (*nad1*, *nad2*, *nad3*, *nad4*, *nad4L*, *nad5*, *nad6*, *nad7*, and *nad9*), three cytochrome *c* oxidase genes (*cox1*, *cox2*, and *cox3*), one transport membrane protein gene (*mttB*), one maturases gene (*matR*), and one cytochrome *b* gene (*cob*). On the other hand, the 15 unique variable genes include four large subunits of ribosomal protein (*rpl2*, *rpl5*, *rpl10*, and *rpl16*), nine small subunits of ribosomal protein (*rps1*, *rps3*, *rps4*, *rps7*, *rps10*, *rps12*, *rps13*, *rps14*, *rps19*), and two succinate dehydrogenases (*sdh3* and *sdh4*). Additionally, we have identified and annotated six plastid-derived PCGs, namely *petG*, *petL*, *psbE*, *psbF*, *psbJ*, and *rpl14*. It is worth mentioning that certain genes exhibit two copies, such as *ccmFN* and *rps19*.

**Table 1:** Gene composition in the mitogenome of *Viburnum chinshanense*

Group of genes	Name of genes
ATP synthase	*atp*1, *atp*4, *atp*6, *atp*8, *atp*9
NADH dehydrogenase	*nad*1, *nad*2, *nad*3, *nad*4, *nad*4L, *nad*5, *nad*6, *nad*7, *nad*9
Cytochrome *b*	*cob*
Cytochrome *c* biogenesis	*ccm*B, *ccm*C, *ccm*FC, *ccm*FN (× 2)
Cytochrome *c* oxidase	*cox*1, *cox*2, *cox*3
Maturases	*mat*R
Transport membrane protein	*mtt*B
Succinate dehydrogenase	*sdh*3*, sdh*4
Ribosomal protein large subunit	*rpl2, rpl*5, *rpl*10, *rpl*16
Ribosomal protein small subunit	*rps*1, *rps*3, *rps*4, *rps*7, *rps*10, *rps*12, *rps*13, *rps*14, *rps*19 (× 2)
Plastid-derived PCGs	*petG, petL, psbE, psbF, psbJ, rpl14*
Ribosome RNA	*rrn5*, *rrn18*, *rrn26*
Transfer RNA (Bacteria-derived)	*trnC-GCA*
Transfer RNA (Plastid-derived)	*trnD-GUC*, *trnH-GUG*, *trnM-CAU*, *trnN-GUU*, *trnP-UGG*, *trnW-CCA*
Transfer RNA (Mitochondrion-native)	*trnC-GCA*, *trnE-UUC*, *trnF-GAA*, *trnG-GCC*, *trnK-UUU*, *trnI-CAU* (× 2), *trnfM-CAU* (× 2), *trnP-UGG*, *trnQ-UUG*, *trnS-GCU*, *trnS-UGA*, *trnY-GUA*
Transfer RNA (unknown)	*trnK-UUU* (× 3), *trnR-UCU*, *trnS-GCU*, *trnS-GGA-1*, *trnS-GGA-2*

The numbers in parentheses represent the number of copies of the gene

We have annotated a total of 28 tRNA genes in the *V. chinshanense* mitogenome, out of which 25 are unique. Among these, 14 tRNA genes are native to the mitochondria. Additionally, we identified six tRNA genes that are derived from the plastid: *trnD-GUC*, *trnP-UGG*, *trnW-CCA*, *trnH-GUG*, *trnN-GUU* and *trnM-CAU*. Notably, we have also discovered a tRNA gene of bacterial origin, *trnC-GCA*. The remaining tRNA genes do not share sequence homology with known organelle tRNA genes and thus their origins remain unknown [29, 30].

Additionally, we have identified three unique rRNA genes in the *V. chinshanense* mitogenome, namely *rrn5*, *rrn18*, and *rrn26*. The specific locations of each gene can be found in Table S1. Figure 2 presents the mitogenome maps of *V. chinshanense*. Among all the annotated genes, 10 PCGs contain introns (Table S1). Specifically, the genes *ccmFC*, *cox2*, *rpl2*, *rps3*, and *rps10* have one intron each, while *nad4* contains three introns. The genes *nad1*, *nad2*, *nad5*, and *nad7* exhibit four introns each.

### Repetitive elements

Microsatellites (simple repeat sequences, SSRs) are usually up to 6 bp tandem sequences in eukaryotic genomes. In the mitogenome of *V. chinshanense*, we have identified a total of 212 SSRs (Table S2). Among these SSRs, tetrameric repeats dominate, accounting for 41.04% (87) of the total count. This is followed by dimeric repeats (49), monomeric repeats (35), trimeric repeats (24), pentameric repeats (12), and hexametric repeats (5). Additionally, we have detected 19 long tandem repeat elements (Table S3).

**Fig. 3:**
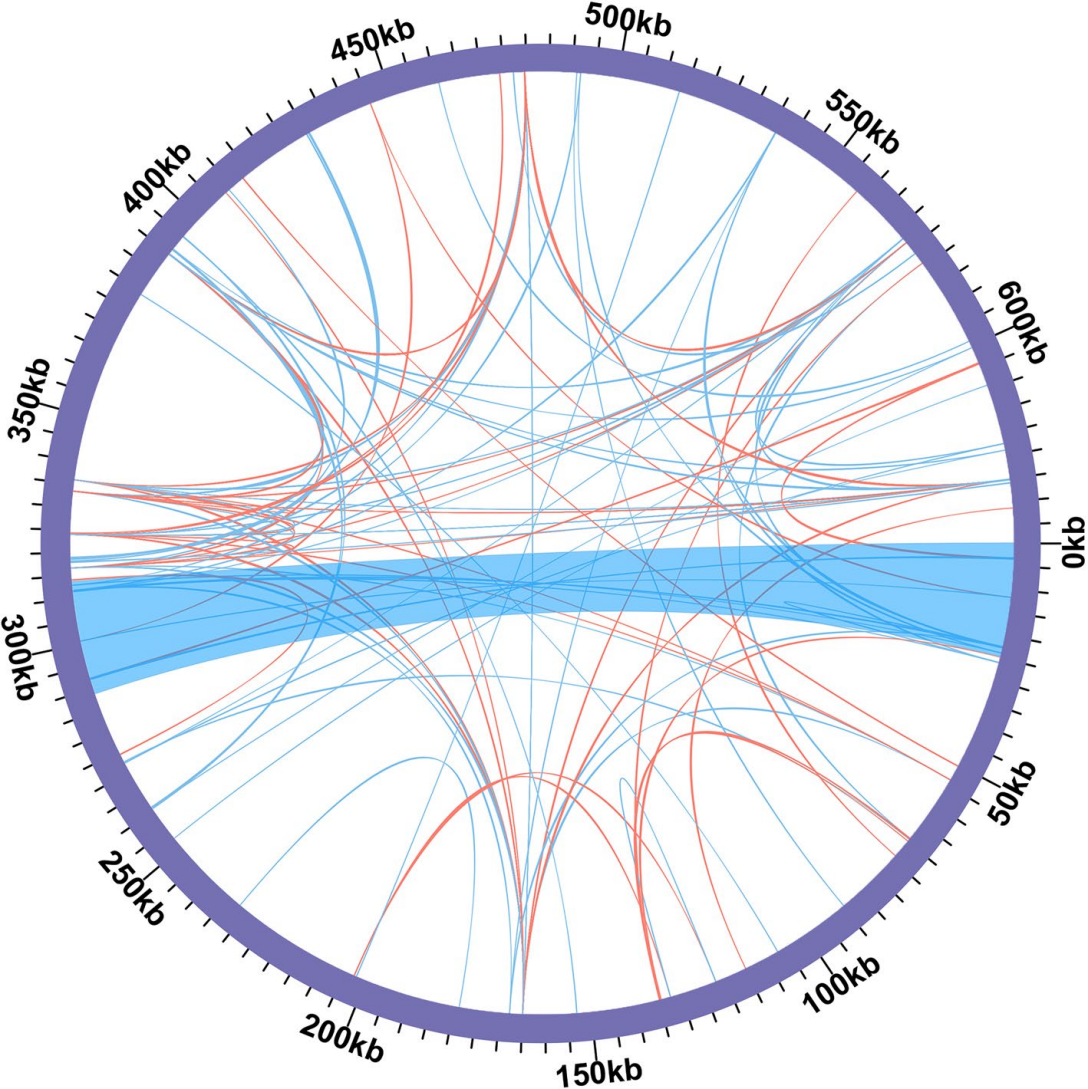
The dispersed repeats identified in the mitogenomes of *V. chinshanense.* The Dispersed repeats (≥ 50 bp) were identified using ROUSFinder. The blue ribbons represent the forward repeats and the red ribbons represent the palindromic repeats. The detailed information about dispersed repeats can be found in Table S4.

Furthermore, we have identified 325 pairs of dispersed repeats in the *V. chinshanense* mitogenome with lengths greater than or equal to 50 bp. These include 169 pairs of forward repeats and 156 pairs of palindromic repeats (Table S4). The number of dispersed repeats is obviously higher than that of SSRs. Most of these repeat elements were less than 500 bp in length. We have detected a long repetitive sequence with length of 24,952 bp, which is the only dispersed repeat in the mitogenome with a length exceeding 1,000 bp. This repeated element corresponds to contig3 in the assembly result. This long repetitive sequence contains two complete genes, namely *ccmFN* and *trnI-CAU*, indicating that there are two copies of each gene. In total, the length of these dispersed repeats amounts to 68,289 bp, accounting for 10.60% of the entire mitogenome of *V. chinshanense*. We have visualized the dispersed repeats of the *V. chinshanense* mitogenome using the Circos (v1.120) package [31] (Fig. 3).

### Characteristic of mitochondrial plastid DNA sequences (MTPTs)

The mitogenome of higher plants often undergoes extensive sequence migration from its plastome and even from nuclear genomes. In our study, we have also annotated the plastidial genome of *V. chinshanense* and compared it with its mitogenome. Using the BLASTn [32] program, we identified a total of 21 mitochondrial-plastid DNA transfers (MTPTs) between the two organelle genomes. These 21 MTPTs have a combined length of 9,902 bp, accounting for 1.54% of the mitogenome (Table S5). Among these MTPTs, MTPT1, MTPT2, MTPT3, and MTPT4 exceed 1,000 bp in length. MTPT1 is the longest, spanning 2,826 bp, while MTPT20 and MTPT21 are the shortest, with only 31 bp. Then we annotated these MTPTs and discovered that most of them contain plastidial genes. As shown in Table S5, MTPT1 harbors a set of plastid genes, primarily related to the photosystem II protein complex, including *psbJ*, *psbL*, *psbF*, *psbE*, and cytochrome b6/f complex subunits (*petL* and *petG*). Additionally, two tRNA genes, *trnW-CCA* and *trnP-UGG*, have migrated along with MTPT1. The plastid gene *rpl14* is located on MTPT4, while the plastid *trnD-GUC* is situated on MTPT7. These two genes also retain some flanking regions within the migrating fragment. In contrast, the remaining three intact tRNA genes (*trnH-GUG*, *trnN-GUU*, and *trnM-CAU*) occupy the entire length of the MTPT, with minimal retained flanking regions after migration.

**Fig. 4:**
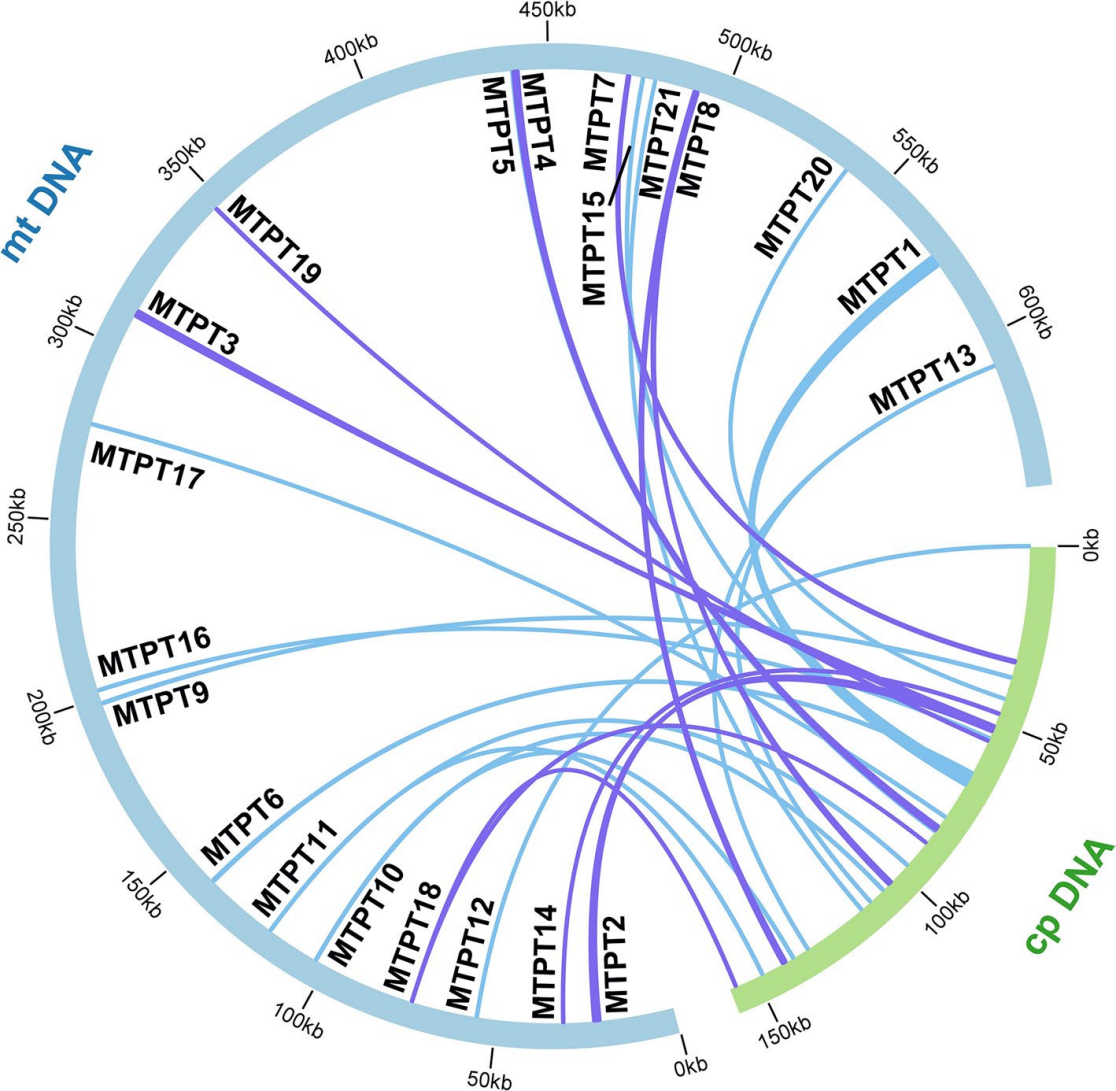
Schematic representation of the distribution of MTPTs between the nineteen mitogenome chromosomes and the plastome of *V. chinshanense***.** The MTPTs on the chloroplast IR regions were counted only once. Different colors of ribbons represent different identities: purple: 70%-90%, and blue: over 90%. The length of each MTPT and the genes it contains can be found in Table S5.

Moreover, we have also identified several gene fragments from plastid migration, including *ndhJ*, *ndhK*, *ndhC*, *rpl16*, and so on. These gene fragments might have experienced sequence loss during the migration process. A schematic representation of the MTPTs is provided in Fig. 4.

### Prediction of RNA editing sites

**Fig. 5:**
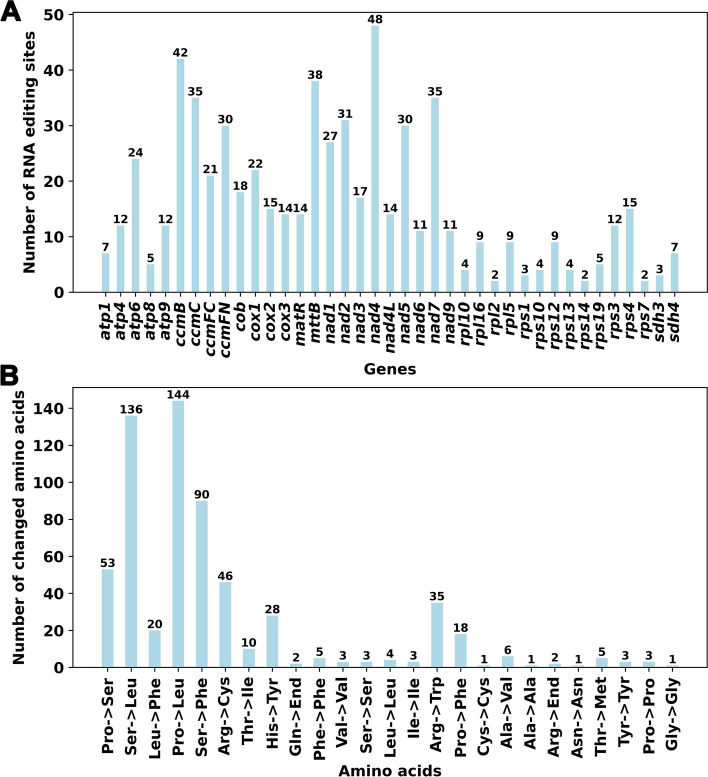
Characteristics of the RNA editing sites identified in mitochondrial PCGs of *V. chinshanense. ***A **The ordinate shows the number of RNA editing sites identified in PCGs, the abscissa shows the name of PCGs identified in the mitogenome of *V. chinshanense*. Panel **B** shows the potential effect of all RNA editing events on amino acids. Most of these RNA editing events lead to changes in amino acids

Using Deepred-mt, we have successfully identified a total of 623 high-confidence C to U RNA editing sites across 39 mitochondrial PCGs (Table S6). The predicted RNA editing sites for each gene can be observed in Fig. 5A. Among these mitochondrial genes, *nad4* exhibits the highest number of RNA editing sites with 48, followed by *ccmB* with 42, making them the top two genes in terms of RNA editing sites. Additionally, *mttB*, *ccmC*, *nad7*, *nad2*, *ccmFN* and *nad5* all possess more than 30 editing sites. On the other hand, genes such as *rps14*, *rpl2*, and *rps7* have the fewest editing sites, with only two C to U edits detected.

According to the findings from Deepred-mt, we have identified C to U RNA editing events in four genes that result in the generation of premature stop codons. These genes include *atp6* (CAA to UAA) and three others (*rps10*, *atp9*, and *ccmFC*) where the CGA codon is edited to UGA. Additionally, the start codons of *rps10* and *nad4L* are created through RNA editing by converting ACG to AUG.

Figure 5B illustrates the impact of RNA editing events on the translated amino acids. A total of 595 RNA editing sites lead to non-synonymous substitutions, and resulting in changes in encoded amino acid, accounting for 95.5% of all. The most common changes involve the replacement of proline (Pro) and serine (Ser) with leucine (Leu), occurring 144 and 136 times, respectively. Furthermore, serine (Ser) is frequently replaced by phenylalanine (Phe) in 90 instances. Collectively, these three substitutions account for 59.4% of the total observed changes. In contrast, there are only 28 synonymous substitutions, indicating that these widespread RNA editing events play a crucial role in protein translation.

### Collinear analysis

**Fig. 6:**
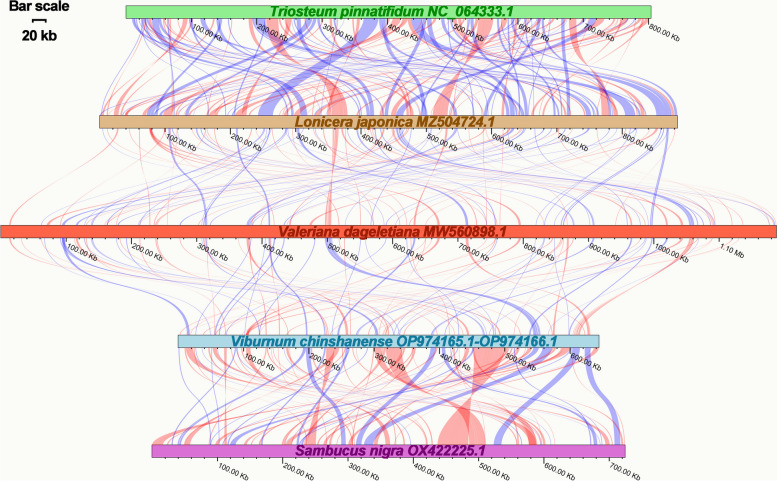
Collinear analysis of *V. chinshanense* mitogenome and its related species. The colorful bars indicated the mitogenomes, and the ribbons showed the homologous sequences between the adjacent species. The blue ribbons indicate regions with homology and the red ribbons indicate where the inversion occurred. The homologous blocks less than 0.5 kb in length are not remaining, and regions that fail to have a homologous block indicate that they are unique to the species. The bar scale showed on the top left

To explore the rearrangements and conserved sequence blocks within the mitogenomes, we employed the BLASTn program to identify homologous collinear blocks. As depicted in Fig. 6, each ribbon connecting two adjacent mitogenomes represents a highly homologous collinear block or sequence. When comparing *V. chinshanense* and *S. nigra* (both belonging to the Viburnaceae family), we observed two large adjacent collinear blocks, measuring 21 kb and 18 kb in length, respectively (Table S7). Additionally, we identified six collinear blocks exceeding 10 kb in length between these two mitogenomes. However, when comparing *V. chinshanense* with *Valeriana dageletiana* of the Caprifoliaceae family, no long collinear blocks were detected, and the longest detected block was only 3 kb in length. Overall, although collinear blocks between closely related species tend to be longer, the mitogenomes exhibited poor collinearity, with numerous regions lacking homology. These findings indicate extensive genomic rearrangement between *V. chinshanense* and related mitogenomes, suggesting that the genomic structure of the mitogenomes is not conserved.

### Gene gain and loss

**Fig. 7:**
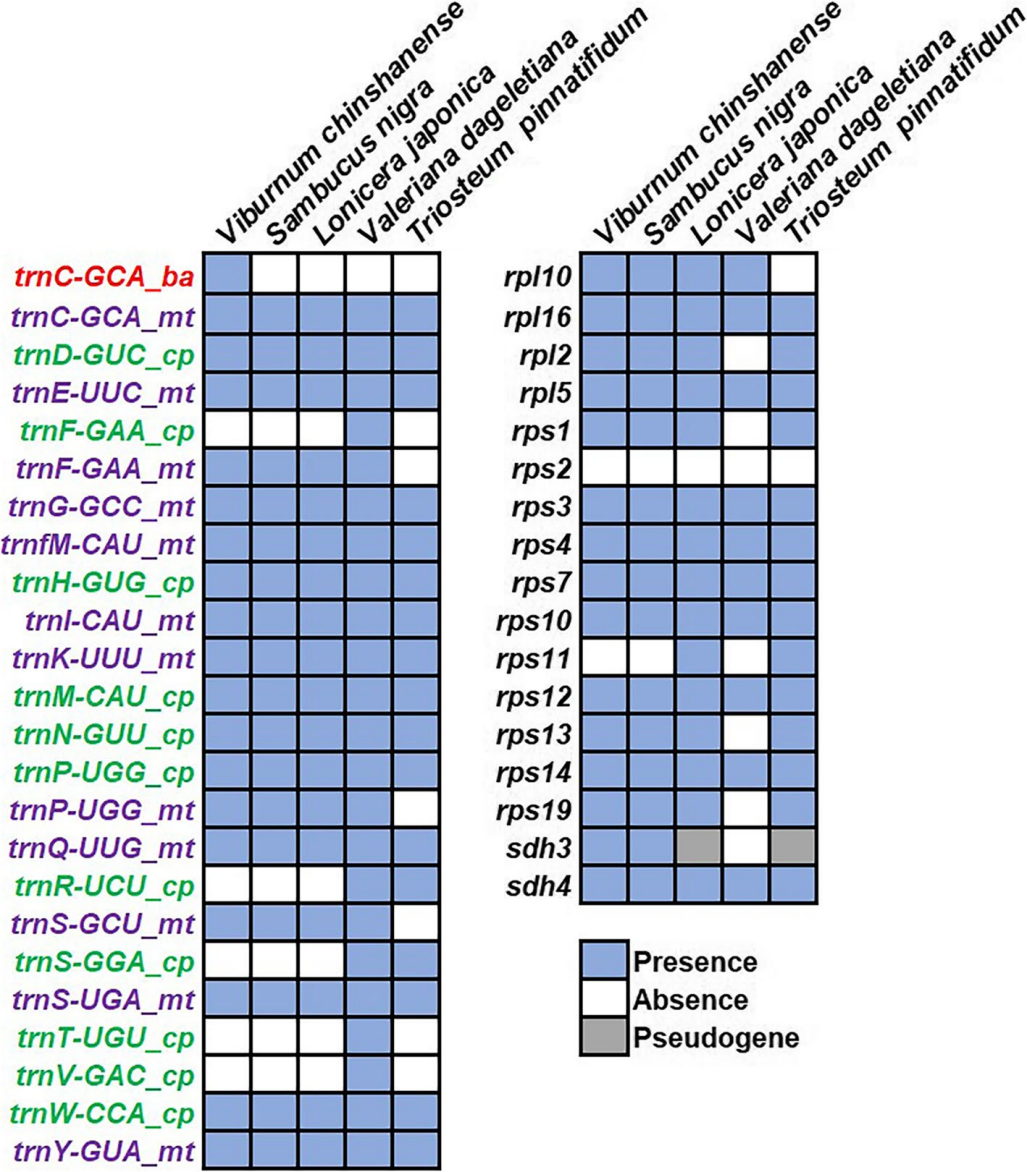
Statistics of mitochondrial variable genes and tRNA genes in 5 Dipsacales species. Light blue squares represent the gene presence of at least one complete copy, white squares represent absence, and gray represents the presence of a fragment of the gene. ‘cp’ represents plastid-derived tRNA genes, ‘ba’ represents bacteria-derived tRNA genes, and ‘mt’ represents mitochondrion-native tRNA genes

To investigate gene losses and gains in the mitogenomes of Dipsacales, we conducted a gene content analysis of *V. chinshanense* along with four others downloaded Dipsacales species. The results are presented in Fig. 7. Initially, it is worth noting that all the analyzed species shared 24 core mitochondrial genes and three unique rRNA genes. Therefore, we did not include these results in Fig. 7. However, the variable genes, on the other hand, exhibit variations in their presence or absence among the species, indicating gene losses or gains between them.

When examining the tRNA genes, we conducted a comprehensive analysis of their origins based on previous studies [19] and sequence similarity. Firstly, it is observed that the number of native mitochondrial tRNA genes is generally conserved across most species. Four species exhibit a total of 12 native mitochondrial tRNA genes, except for *T. pinnatifidum*, which has only 9. In the mitogenome of *T. pinnatifidum*, *trnF-GAA*, *trnP-UGG*, and *trnS-GUC* are absent. Additionally, we have identified 11 tRNA genes in the Dipsacales mitogenomes that have originated from plastids and migrated to varying extents. Among these, six tRNA genes (*trnD-GUC*, *trnH-GUG*, *trnM-CAU*, *trnN-GUU*, *trnP-UGG*, and *trnW-CCA*) are present in all five mitogenomes analyzed. In contrast, plastid-derived *trnF-GAA*, *trnT-UGU*, and *trnV-GAC* are exclusively found in *Valeriana dageletiana*, while *trnR-UCU* and *trnS-GGA* are found in *V. dageletiana* and *T. pinnatifidum*. Furthermore, three native mitochondrial tRNA genes identified in other angiosperms (*trnD-GUC*, *trnV-UAC*, and *trnW-CCA*) are absent in all five Dipsacales mitogenomes. Moreover, among the five species analyzed, only *V. chinshanense* harbors a bacteria-derived tRNA gene, namely *trnC-GCA*.

Among the variable protein-coding genes (PCGs), there are only nine genes that are fully shared among the five mitogenomes. These genes are *rpl16*, *rpl5*, *rps3*, *rps4*, *rps7*, *rps10*, *rps12*, *rps14* and *sdh4*. However, the remaining genes have experienced varying degrees of gene loss. Notably, the gene *rps2* has been completely lost in all the analyzed species.

### Phylogenetic analysis

**Fig. 8:**
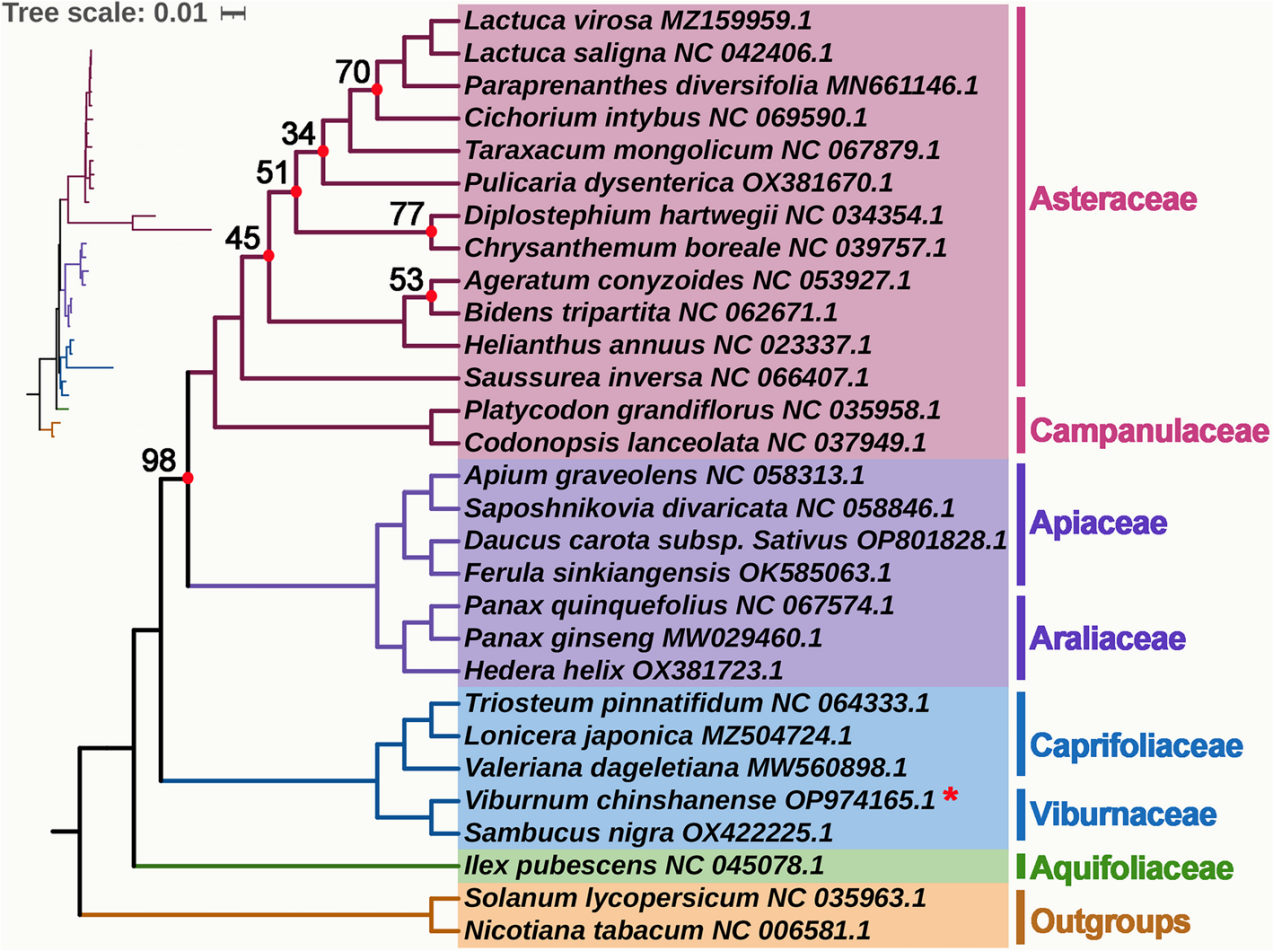
The phylogenetic relationships of *V. chinshanense* and another 28 species based on conserved mitochondrial genes*.* The tree was constructed based on the nucleotide sequences of 27 conserved mitochondrial protein-coding genes (PCGs), including *atp*1, *atp*4, *atp*6, *atp*8, *atp*9, *ccm*B, *ccmC*, *ccmFC*, *ccmFN*, *cob*, *cox*1*, cox*2, *cox*3, *matR*, *mttB*, *nad*1, *nad*2, *nad*3, *nad*4, *nad*4*L*, *nad*5, *nad*6, *nad*7, *nad*9, *rps*3, *rps*12, and *sdh4*. We used Maximum Likelihood (ML) method to reconstruct the phylogenetic tree. The ML topology is indicated with ML bootstrap support values. *Amborella trichopoda* was used as an outgroup. The species list and its accession numbers that used in phylogenetic analysis are shown in Table S8

We conducted a phylogenetic analysis using 29 mitogenomes of angiosperm species, with *Nicotiana tabacum* and *Solanum lycopersicum* serving as outgroups. The species list and corresponding GenBank accessions used for the analysis can be found in Table S8. A total of 27 shared PCGs were aligned and concatenated to create the matrix data. The phylogenetic analysis resulted in a maximum likelihood (ML) tree with robust support along the main basal branches (Fig. 8). In terms of phylogenetic relationships, *V. chinshanense* is most closely related to *S. nigra*, with both species belonging to the Viburnaceae family. Notably, these two species are currently the only ones with available mitogenome data within the Viburnaceae family. The Viburnaceae family is identified as the sister group to the Caprifoliaceae family, with a bootstrap value of 100%. At the family level, the overall phylogenetic tree closely aligns with the APG IV system. However, within the Asteraceae family, some nodes lack high bootstrap support, suggesting that phylogenetic inference based solely on mitochondrial PCGs may not be suitable for resolving lower taxonomic categories.

## Discussion

Our study focuses on analyzing the mitogenome of *V. chinshanense*, which spans a length of 643,971 bp (Fig. 2). It's worth noting that there is a potential for alternative configurations and a multi-chromosomal genome architecture. This was evident when Unicycler utilizing long-reads to resolve repeated regions and generated two independent circular contigs (Fig. 1D). A notable feature is the 24,952 bp repetitive element, which could play a significant role in recombination events. This repetitive element encompasses two mitochondrial genes, namely *ccmFN* and *trnI-CAU*, resulting in the presence of two complete copies of each gene. It is common for plant mitogenomes to undergo recombination facilitated by large repetitive segments dispersed across the mitochondrial DNA. For example, an 16,660 bp long repetitive element were reported in *Photinia serratifolia* [33], accounting for 7% of the mitogenome. A repetitive element spanning 21,809 bp was discovered in the mitogenome of *Taraxacum mongolicum*, and its existence was confirmed through PCR experiments [34]. Interestingly, a total of five pairs of repetitive elements have been identified to play a role in recombination events within dandelion mitogenomes. Furthermore, extensive research has revealed that short dispersed repeats contribute to mitogenome recombination in various plant species, including *Nymphaea colorata* [35], *Silene latifolia* [19], *Scutellaria tsinyunensis* [36], and *Ginkgo biloba* [37], resulting in the emergence of different alternative configurations. The intricate structural variations observed in plant mitogenomes have been the subject of extensive investigation in previous studies [22, 23, 38], and several tools have been developed to unravel these dynamically changing genomes [21, 39]. With the advancements in sequencing technology, high-depth sequencing approaches have the potential to detect low-frequency recombination events. Unfortunately, in our study, we were unable to detect any long-reads that would confirm the presence of recombinant sequences due to length limitations. However, our PCR experiments recognize the correctness of the assembly, indicating the possibility of mitogenome recombination. In the future, obtaining longer reads will be crucial in obtaining direct evidence supporting the existence of multiple genomic configurations mediated by such long repetitive segments.

The unique genome structure and evolutionary processes of plant mitogenomes make them highly susceptible to acquiring and incorporating foreign DNA [40]. Compared to plastid DNA, plant mitogenomes are more inclined to accept and integrate foreign DNA, which is commonly observed [41]. For instance, split and partial gene transfer events involving the ribosomal protein gene *rpl2* have been identified in plant mitogenomes, providing direct evidence for the ease with which foreign DNA can be integrated into these genomes[42]. Recent studies have revealed that approximately 13% of the zucchini mitogenome is composed of sequences originating from plastid and nuclear sources, predominantly non-coding sequences [41]. Another study [43] reported the horizontal transfer of a 356 bp region near the 3' end of the *atp1* gene from Lamiales plants to *Tolypanthus maclurei* mitogenomes. Horizontal gene transfer (HGT) between organellar genomes and the nuclear genome is widespread and plays a significant role in plant evolution. In the case of *V. chinshanense*, we observed a substantial presence of sequences transferred from the plastidial genome to the mitogenome (Table S5, Fig. 4). Among these transferred sequences, MTPT1 is the longest. Previous studies have also reported the existence of large segments of transferred plastid DNA in mitogenomes. For example, in *Suaeda glauca*, 26.87 kb mitochondrial plastid DNA fragments constitute 5.18% of its mitogenome [17]. These large fragments are believed to have profound implications for eukaryotic evolution, promoting genetic diversity. Furthermore, it has been demonstrated that the origin of tRNAs in plant mitochondria is twofold: some are inherited from the mitochondrial ancestor, while others are acquired from chloroplasts through HGT [44].

Based on sequence similarity and previous reports [45], we were able to identify tRNA genes in the *V. chinshanense* mitogenome that were transferred from the plastid to the mitochondria. Specifically, *trnD-GUC* (cp), *trnP-UGG* (cp), *trnW-CCA* (cp), *trnH-GUG* (cp), *trnN-GUU* (cp), and *trnM-CAU* (cp) were potentially acquired from the plastid. In contrast, the mitogenome of *V. chinshanense* contains 12 native tRNA genes, indicating that approximately one-third of the tRNA genes have been horizontally transferred from the plastid. Over time, these transfer events have led to the acquisition of functional tRNAs that are conserved across angiosperms [37, 46, 47]. Among these transferred tRNA genes, *trnW-CCA* (cp) is frequently observed in the mitogenomes of other angiosperms, and it appears to be homologous to its chloroplast counterpart, except in *Amborella trichopoda*, where a native mitochondrial *trnW-CCA* gene is present but absent in other angiosperms [30]. Additionally, a study reported that *trnP-UGG* (cp) is likely functional [45]. Previous studies have suggested the potential functionality of *trnH-GUG* (cp) and *trnM-CAU* (cp) in plant mitogenomes and proposed that they were transferred during an early stage of evolution [41, 48]. Furthermore, we also identified a bacteria-derived tRNA gene, *trnC-GCA*, in the *V. chinshanense* mitogenome. This bacteria-derived *trnC-GCA* has been previously reported [29, 30], although not all angiosperms possess this gene. Its presence has not been detected in the mitogenomes of the other four published species in the Dipsacales.

During the transfer of DNA fragments from chloroplasts to the mitogenome, it is common for some fragments to carry PCGs. However, these transferred genes often become nonfunctional pseudogenes [29]. In our study, we identified a set of intact plastid genes associated with photosynthesis (*psbJ, psbL, psbF, psbE, petL*, and *petG*) in the *V. chinshanense* mitogenome (Table S5). These genes may have been transferred relatively recently, possibly along with the larger fragment (MTPT1). However, it is likely that these transferred genes are nonfunctional in the mitogenome due to the absence of the corresponding enzyme system. The presence of large homologous MTPTs also highlights the importance of avoiding these regions in the genus *Viburnum* when designing molecular markers based on plastid DNA. This precaution is necessary to prevent potential barcode paradoxes [49].

Plant mitochondrial RNA editing is a fascinating biological phenomenon in which specific nucleotide positions within the mitochondrial RNA sequence undergo base mutations catalyzed by mitochondrial RNA editing enzymes [50–52]. These RNA editing enzymes belong to a unique class of deaminases that catalyze the conversion of C to U or U to C in the RNA sequence [53]. In plants, RNA editing plays a crucial role in mitochondrial gene expression and function [54], as many of these RNA editing events can lead to alterations in RNA sequences and variations in the translated protein products [55]. RNA editing of mitochondrial genes is believed to be an important factor in regulating plant cytoplasmic inheritance-related traits [56]. The Plant Editosome Database (https://ngdc.cncb.ac.cn/ped/) has documented 1673 instances of RNA editing in plant organelle genomes, including 1541 instances in plastidial genomes and 132 instances in mitogenomes, underscoring the widespread occurrence of RNA editing sites in plant organelles. In our study, we identified a total of 623 RNA editing events in *V. chinshanense*, with most of the RNA editing sites occurring at the first or second positions, consistent with observations in other plant species [36, 57–60]. The identification of RNA editing sites can provide valuable insights for predicting the functional implications of newly encoded codons. Notably, we discovered that RNA editing events generated stop codons in four genes (*atp6, atp9, rps10,* and *ccmFC*) and start codons in two genes (*rps10* and *nad4L*), and these findings were supported by the high-confidence predictions of Deepred-mt. The emergence of new start and stop codons is often associated with the production of proteins that are highly conserved and homologous to those found in other species, thereby facilitating efficient gene expression in mitochondria [55].

## Conclusion

In this study, we have successfully assembled the mitogenome of *V. chinshanense*, revealing a master circular genome structure. Comprehensive analyses were conducted to investigate its gene content, repetitive elements, RNA editing sites, and other fundamental characteristics, in addition to making phylogenetic inferences. To the best of our knowledge, this is the first comprehensive description of a complete mitogenome within the *Viburnum* genus. Our findings shed new light on the evolutionary dynamics of mitochondrial genes, providing valuable insights into the evolutionary history of mitogenomes in the Dipsacales order.

## Materials and methods

### Plant sampling, DNA extracting and sequencing

We collected fresh leaves of *V. chinshanense* in July 2022 from the Jinfo Mountains (N28°52′, E107°27′). Jie Yu undertook the formal identification of *V. chinshanense.* These specimens have been deposited in the herbarium of Southwest University, Chongqing, China, with the accession number VC20220715. Genomic DNA was extracted using the Tiangen Biotech DNA kit (Beijing). For library construction, we utilized the NEBNext® library building kit with an insert size of 350 bp. The constructed DNA library was sequenced on the NovaSeq 6000 platform at Benagen (Wuhan, China), generating a total of approximately 15 Gb of raw data. To ensure data quality, we applied Trimmomatic to remove low-quality sequences, including those with a quality value (Q) of less than or equal to 5, which accounted for more than 50% of the total bases, as well as sequences containing more than 10% "N" bases. Furthermore, the plant sample used for Illumina sequencing was also subjected to Oxford Nanopore sequencing. Purified DNA was prepared for long-read sequencing following the protocol outlined in the SQK-LSK109 genomic sequencing kit (ONT, Oxford, UK). In total, we obtained 13.96 Gb of raw reads, with N50 and N90 values of 17,365 and 4,382, respectively.

### Organelle genome assembly

For plastome assembly, we utilized GetOrganelle v1.7.4.1 with the following parameters: ‘-R 15 -k 21,45,65,85,105 -F embplant_pt’ to assemble the Illumina short-reads [61]. GetOrganelle generated two complete plastome sequences, and we selected the one where the SSC region aligns in the same direction as *Arabidopsis thaliana*. Subsequently, we performed de novo assembly of *V. chinshanense* long-reads using Flye (v.2.9.1-b1780) with the parameters ‘–min-overlap 2,000’. Flye assembler generated a total of 92,028 sequences, the longest of which was 898,484 bp and Fragments N50 was 68,590 bp. BLASTn [32] was employed to identify the draft mitogenome from the assembled sequences. To achieve this, we constructed a database for the assembled sequences using makeblastdb and used all mitochondrial genes from *Liriodendron tulipifera* (NC_021152.1) as query sequences to identify contigs containing these genes. Three mitochondrial contigs were successfully identified. We then mapped the short-reads and long-reads to these contigs, retaining all mapped reads with the help of BWA and SAMTools [62, 63]. Considering the presence of homologous regions between the mitogenome and chloroplast sequences, it is probable that these regions were replaced by their chloroplast counterparts during the polishing process. To address this, we performed hybrid assembly using Unicycler [64] by combining Illumina short-reads and Nanopore long-reads. The mapped Illumina short-reads were initially assembled using SPAdes [65], and then the Nanopore long-reads were employed to resolve repetitive sequence regions in the assembly, using minimap2 [66]. After multiple iterations and adjustments, we determined the optimal kmer value of 71. A total of 3,279 sequences were obtained by spades assembler, the longest was 240,812 bp in length, and the average length was 523.2 bp. The resulting GFA format files generated by Unicycler were visualized using Bandage [67]. Ultimately, Unicycler produced three contigs with overlapping regions, which aligned with the initial assembly from Flye. Notably, as these contigs were assembled based on Illumina short-reads, no additional polishing steps were necessary.

### Verification of the genome structure

In our study, we employed PCR experiments to investigate the structure of *V. chinshanense*. Specifically, we designed four specific primers targeting four junctions (p1-p4, Fig. 1A) to verify the accuracy of assembly. The primer design was conducted using the Primer designing tool on NCBI (https://www.ncbi.nlm.nih.gov/tools/primer-blast/; accessed on 12 June 2023) with default parameters. The primer sequences used for PCR reactions are listed in Table S9. Subsequently, DNA was extracted, and the amplifications were performed using a Pro-Flex PCR system (Applied Biosystems, Waltham, MA, USA). The PCR reaction volume was 25 µL, comprising 2 µL of template DNA, 1 µL of forward primer, 12.5 µL of 2 × Taq PCR Master Mix, and 9.5 µL of ddH2O. The amplification conditions consisted of an initial denaturation at 94 ℃ for 5 min, followed by 30 cycles of denaturation at 94 ℃ for 30 s, annealing at 58 ℃ for 30 s, extension at 72 ℃ for 60 s, and a final extension step at 72 ℃ for 5 min. The PCR amplicons were visualized using 1% agarose gel electrophoresis. Subsequently, the single bright bands were excised and sent to Sangon Biotech (Shanghai, China) Co., Ltd. for Sanger sequencing.

### Mitogenome and plastidial genome annotation

The plastome of *V. chinshanense* was annotated using CPGAVAS2 [68] with the plastomes of *V. farreri* (NC_056112.1) and *V. schensianum* (NC_056104.1) serving as reference genomes. The annotation results were further verified using CPGView [69] to ensure accurate gene annotations.

For the annotation of the assembled mitogenome of *V. chinshanense*, we utilized GeSeq [70] with four reference mitogenomes from GenBank. The first reference mitogenome was that of *Liriodendron tulipifera* (NC_021152.1), which is an angiosperm with the most comprehensive mitochondrial gene annotations. The remaining three reference mitogenomes were *Triosteum pinnatifidum* (NC_064333.1), *Sambucus nigra* (OX422225.1), and *Lonicera japonica* (MZ504724.1). These three species are the most closely related to Viburnum and all belong to the Order Dipsacales. The tRNA annotations were performed using tRNAscan-SE [71]while rRNA annotations were obtained through BLASTn [32]. To ensure accuracy, manual edits were made to the annotations using Apollo [72]. Finally, the genome map was generated using OGDRAW (version 1.3.1) [41].

### Repetitive elements

The long tandem repeats were detected by Tandem Repeats Finder (TRF, https://tandem.bu.edu/trf/trf.html) with the default parameters. The simple sequence repeats (SSRs) of the assembled mitogenome were identified using the online website MISA (https://webblast.ipk-gatersleben.de/misa/), the parameters of the minimum numbers of mono-, di-, tri-, tetra-, penta-, and hexanucleotides were set as 10, 5, 4, 3, 3, and 3, respectively. Additionally, forward, reverse, palindromic, and complementary repeat sequences were identified using REPuter [73] (https://bibiserv.cebitec.uni-bielefeld.de/reputer/) with the following settings: hamming distance of three and minimal repeat size of 30 bp, and e-value is limited to less than 1*e*-05. The visualization of the repetitive elements was done using the Circos package [74].

### Identification of the mitochondrial plastid sequences (MTPTs)

To identify the mitochondrial plastid sequences (MTPTs), we compare the plastome and mitogenome sequences of *V. chinshanense* by using BLASTn [32] program with the following parameters: -evalue 1*e*-5, -word_size 9, -gapopen 5,—gapextend 2, -reward 2, -penalty -3. The BLASTn [32] results were visualized using Circos package [74]. The identified MTPTs were also annotated by using GeSeq. For the two MTPTs located on the inverted repeat regions of the plastome, we count only one time.

### Prediction of RNA editing sites

We used Deepred-mt [75], a convolutional neural network (CNN) model-based tool, to predict C to U RNA editing sites in the mitochondrial genome. To perform the prediction, we extracted all mitochondrial protein-coding genes from the mitogenome and inputted them into the Deepred-mt tool. We considered predictions with probability values greater than 0.9 as reliable results.

### Collinear analysis

For the collinear analysis with *V. chinshanense*, we selected *Liriodendron tulipifera* (NC_021152.1) as well as three closely related species: *Triosteum pinnatifidum* (NC_064333.1), *Sambucus nigra* (OX422225.1), and *Lonicera japonica* (MZ504724.1). We identified collinear blocks based on sequence similarity using the BLASTn [32] program with the following parameters: -evalue 1*e*-5, -word_size 9, -gapopen 5,—gapextend 2, -reward 2, -penalty -3. Only collinear blocks longer than 1 kb were retained for downstream analysis. To visualize the collinear relationships, we generated a multiple synteny plot using LINKVIEW2 (https://github.com/YangJianshun/LINKVIEW2).

### Phylogenetic analysis

We retrieved a total of 29 mitogenomes, including two outgroups (*Nicotiana tabacum* and *Solanum lycopersicum*), from the NCBI nucleotide database (https://www.ncbi.nlm.nih.gov/) These mitogenomes were used to construct a phylogenetic tree with *V. chinshanense*. Firstly, PhyloSuite (v.1.2.2) [76] was employed to identify and extract 27 orthologous protein-coding genes (PCGs) across the analyzed species. The nucleotide sequences corresponding to these PCGs were then aligned using MAFFT (v7.471) [77]. Subsequently, the aligned sequences were concatenated to generate the input for phylogenetic tree construction. The maximum likelihood (ML) method was implemented using IQ-TREE (version 2.1.4-beta) [78] with the parameters “–alrt 1000 -B 1000”. The bootstrap analysis was performed with 1,000 replicates. Finally, the resulting phylogenetic tree was visualized and edited using the online tool ITOL [79].

### Supplementary Information

**Additional file 1: Figure S1. **The full alignment of Sanger sequencing reads. p1, p2, p3, and p4 represent path 1, path 2, path 3 and path 4 respectively.

**Additional file 2:** **Figure S2**.The original uncut electropherogram.

**Additional file 3:** **Table S1. **The detailed location of the annotated genes in *V. chinshanense* mitogenome. **Table S2. **The identified simple sequence repeats (SSRs) in *V. chinshanense* mitogenome. **Table S3 **The identified long tandem repeats in *V. chinshanense* mitogenome. **Table S4. **The identified dispersed repeats in *V. chinshanense* mitogenome. **Table S5. **The identified mitochondrial plastid sequences (MTPTs) in *V. chinshanense* mitogenome. **Table S6. **The identified RNA editing sites in protein-coding genes of *V. chinshanense* mitogenome. **Table S7. **Colinear analysis among *V. chinshanense* mitogenome and four related mitogenomes. **Table S8. **Species list used for phylogenetic analysis in this study. **Table S9. **Primers used uses in this stud.

## Data Availability

The plastome and mitogenome of *V. chinshanense* sequences are available in the nucleotide database of NCBI (https://www.ncbi.nlm.nih.gov/), with the accession numbers: OP994186.1 and OP974165.1-OP974166.1. The mitochondrial reads used for mitogenome assembly in this study have been released on the NCBI with those accession numbers: PRJNA941136 (BioProject); SAMN33591000 (BioSample), SRR23703831 and SRR23703832 (SRA).

## Abbreviations

SSR: Simple sequence repeat
ML: Maximum likelihood
NCBI: National Center for Biotechnology Information
BLAST: Basic Local Alignment Search Tool
PCGs: Protein-coding gene sequences
MTPT: Mitochondrial plastid DNA
WGS: Whole genome sequencing
HGT: Horizontal Gene Transfer
