# Supplementary material for: Assembly and comparative analysis of the complete mitochondrial genome of Viburnum chinshanense
Source: BMC Plant Biol. 2023 Oct 11;23:487. doi: 10.1186/s12870-023-04493-4 (PMC10566092; doi:10.1186/s12870-023-04493-4)
Supplement: Supplementary file 1 — Additional file 1: Figure S1. The full alignment of Sanger sequencing reads. p1, p2, p3, and p4 represent path 1, path 2, path 3 and path 4 respectively. [file 12870_2023_4493_MOESM1_ESM.docx]

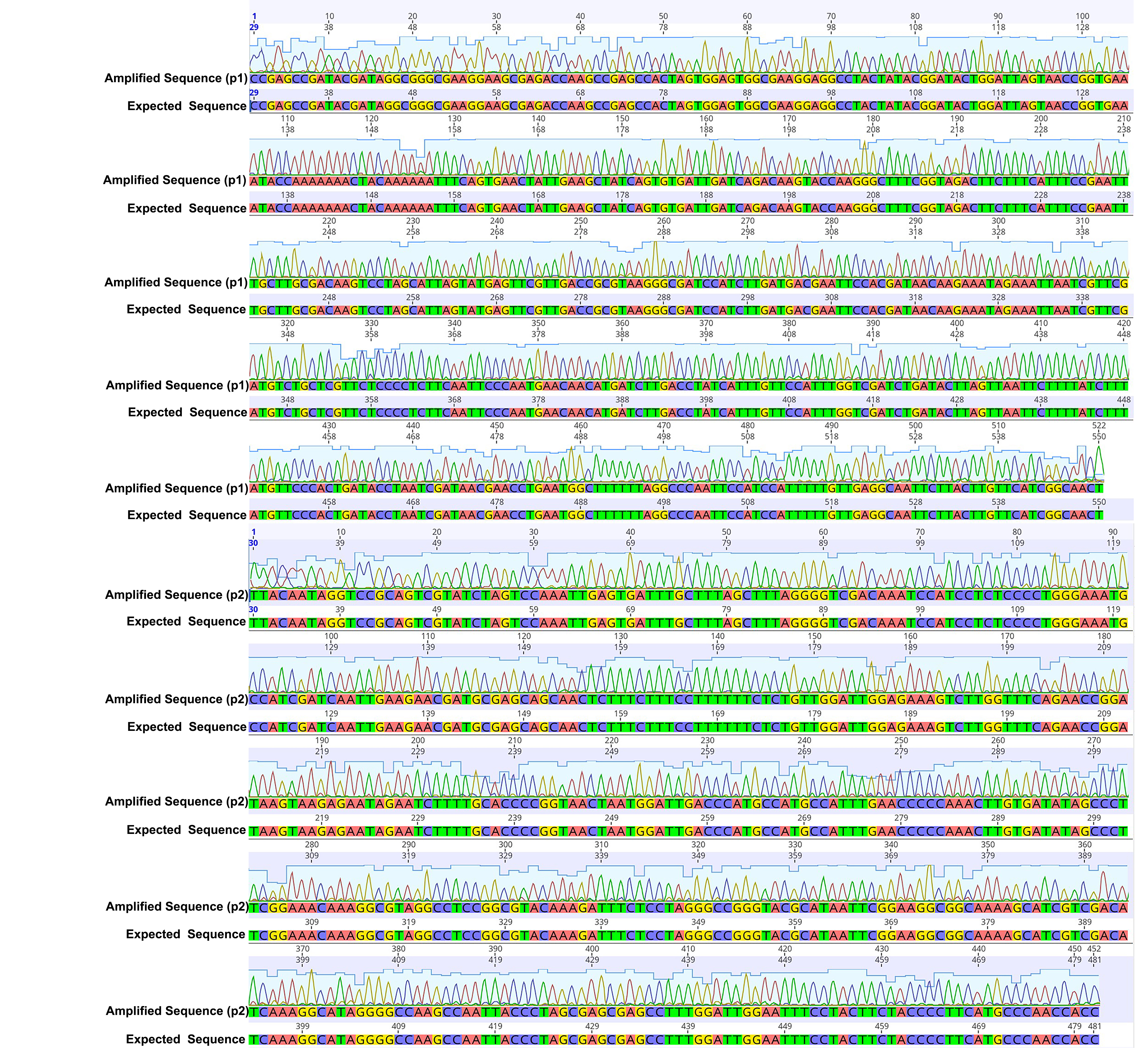


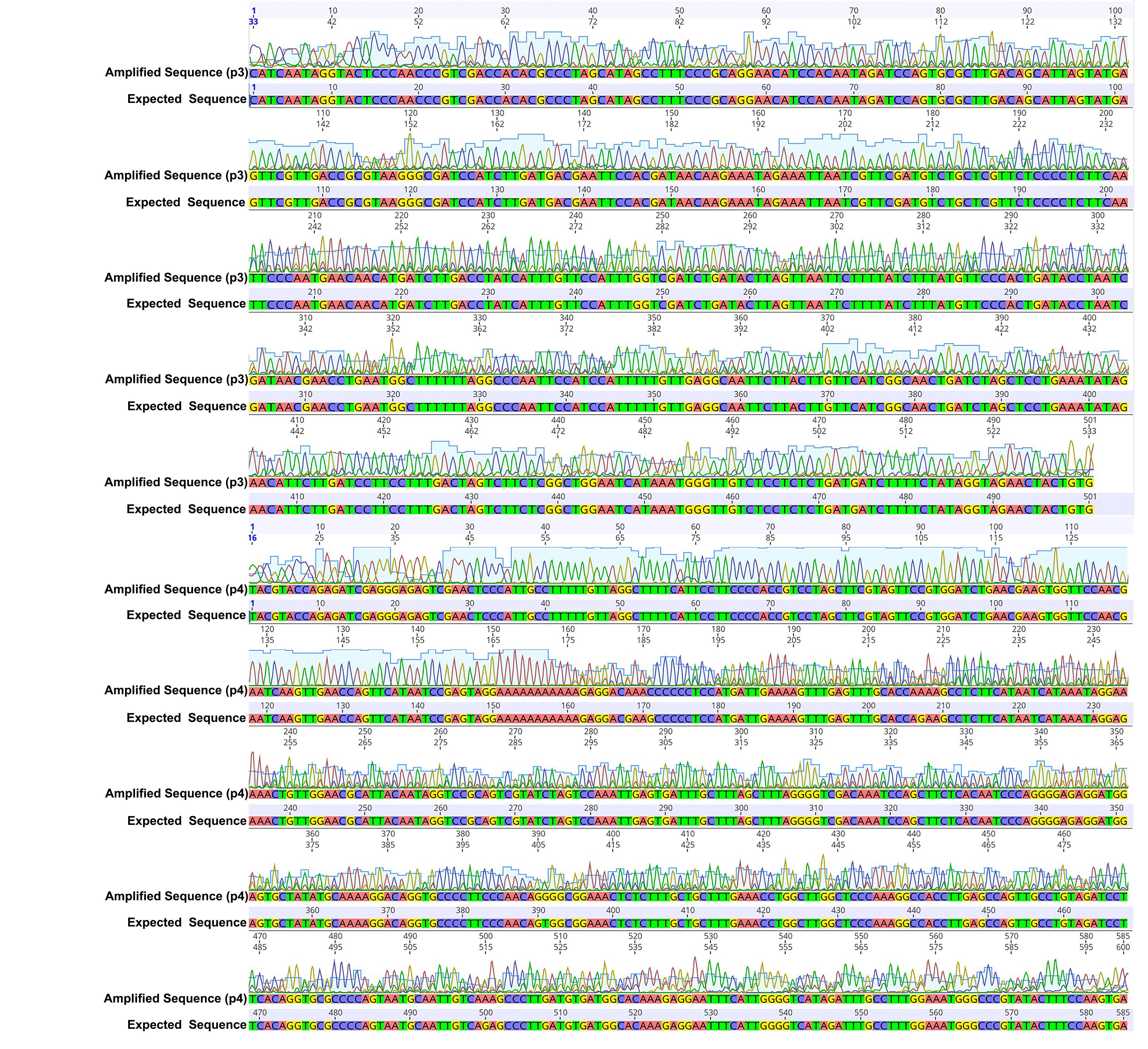


**Figure S1. The full alignment of Sanger sequencing reads.**

p1, p2, p3, and p4 represent path 1, path 2, path 3 and path 4 respectively.


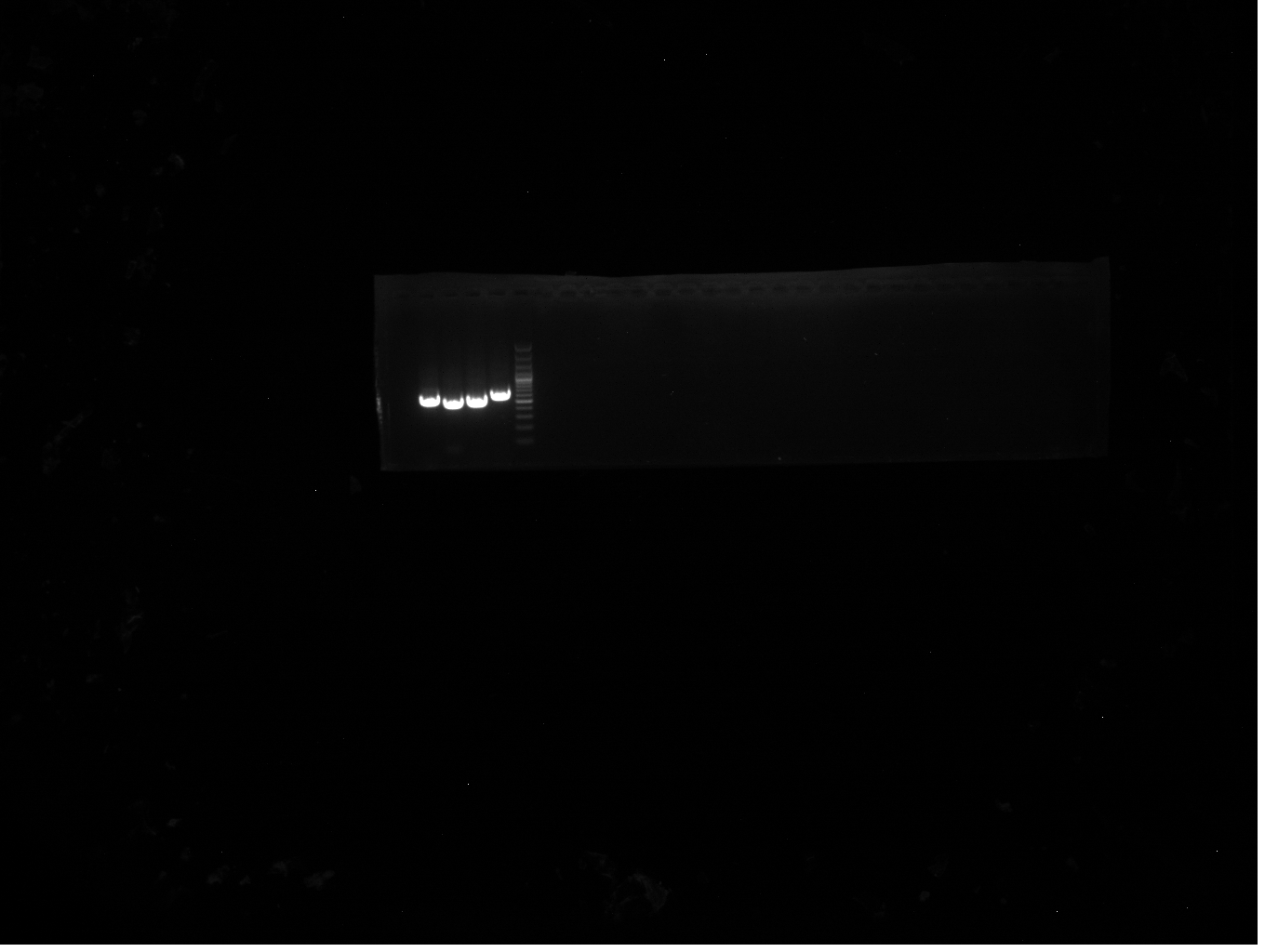


**Figure S2.** **The original uncut electropherogram.**
