# Supplementary figures and images for: Assembly and comparative analysis of the complete mitochondrial genome of Viburnum chinshanense
Source: BMC Plant Biol. 2023 Oct 11;23:487. doi: 10.1186/s12870-023-04493-4 (PMC10566092; doi:10.1186/s12870-023-04493-4)

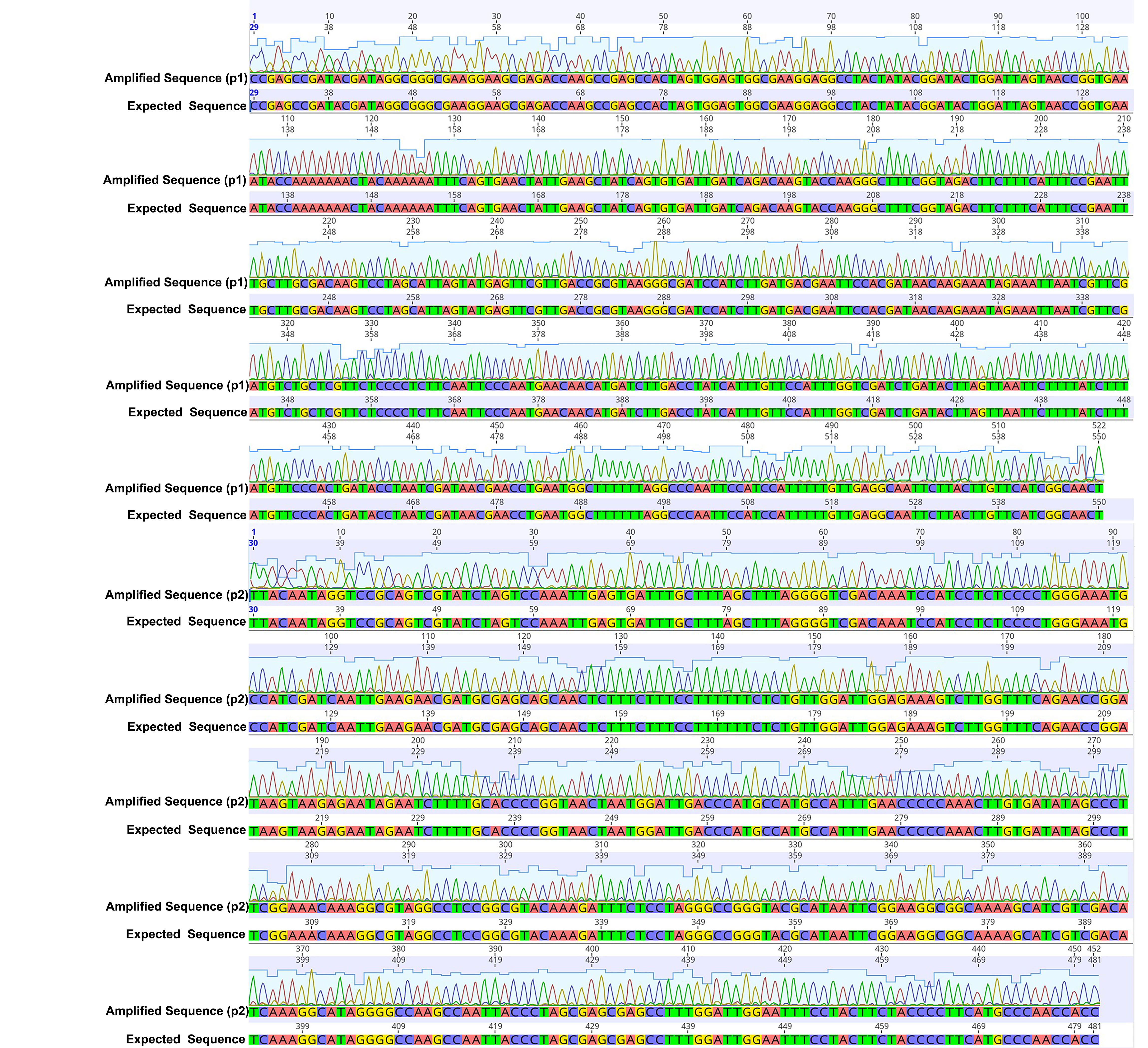

Supplement: Supplementary file 2 — Additional file 2: Figure S2.The original uncut electropherogram. [file 12870_2023_4493_MOESM2_ESM.jpg]

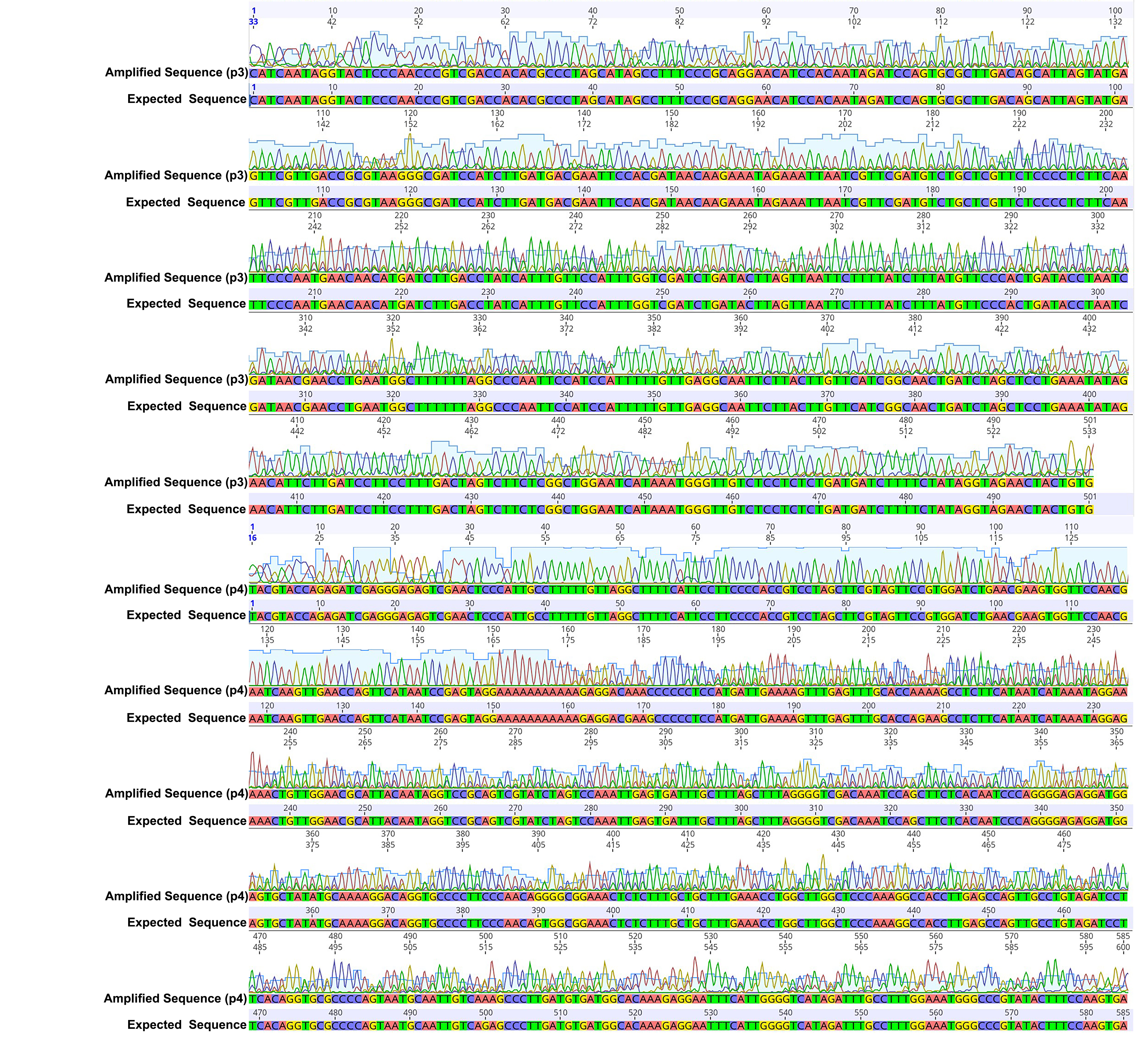

Supplement: Supplementary file 3 — Additional file 3: Table S1. The detailed location of the annotated genes in V. chinshanense mitogenome. Table S2. The identified simple sequence repeats (SSRs) in V. chinshanense mitogenome. Table S3 The identified long tandem repeats in V. chinshanense mitogenome. Table S4. The identified dispersed repeats in V. chinshanense mitogenome. Table S5. The identified mitochondrial plastid sequences (MTPTs) in V. chinshanense mitogenome. Table S6. The identified RNA editing sites in protein-coding genes of V. chinshanense mitogenome. Table S7. Colinear analysis among V. chinshanense mitogenome and four related mitogenomes. Table S8. Species list used for phylogenetic analysis in this study. Table S9. Primers used uses in this stud. [file 12870_2023_4493_MOESM3_ESM.jpg]
